# Nrf2: a dark horse in doxorubicin-induced cardiotoxicity

**DOI:** 10.1038/s41420-023-01565-0

**Published:** 2023-07-26

**Authors:** Xiaopeng Zhao, Zheng Tian, Mingli Sun, Dan Dong

**Affiliations:** 1 College of Exercise and Health, Shenyang Sport University, Shenyang, Liaoning 110102 China; 2 Department of Pathophysiology, College of Basic Medical Science, China Medical University, Shenyang, Liaoning 110122 China

**Keywords:** Physiology, Cardiovascular diseases

## Abstract

Being a broad-spectrum anticancer drug, doxorubicin is indispensable for clinical treatment. Unexpectedly, its cardiotoxic side effects have proven to be a formidable obstacle. Numerous studies are currently devoted to elucidating the pathological mechanisms underlying doxorubicin-induced cardiotoxicity. Nrf2 has always played a crucial role in oxidative stress, but numerous studies have demonstrated that it also plays a vital part in pathological mechanisms like cell death and inflammation. Numerous studies on the pathological mechanisms associated with doxorubicin-induced cardiotoxicity demonstrate this. Several clinical drugs, natural and synthetic compounds, as well as small molecule RNAs have been demonstrated to prevent doxorubicin-induced cardiotoxicity by activating Nrf2. Consequently, this study emphasizes the introduction of Nrf2, discusses the role of Nrf2 in doxorubicin-induced cardiotoxicity, and concludes with a summary of the therapeutic modalities targeting Nrf2 to ameliorate doxorubicin-induced cardiotoxicity, highlighting the potential value of Nrf2 in doxorubicin-induced cardiotoxicity.

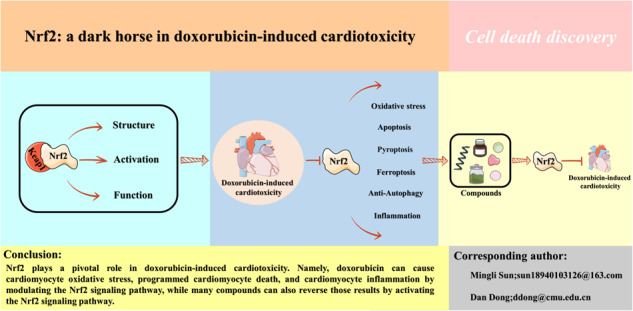

## Facts

1. The pathological mechanisms of doxorubicin-induced cardiotoxicity are complex.
2. Nrf2 is involved in multiple pathological mechanisms of doxorubicin-induced cardiotoxicity, especially in cell death as well as oxidative stress.
3. Targeting Nrf2 may help to mitigate doxorubicin-induced cardiotoxicity.

## Open questions

1. Which of these are specifically included in the pathological mechanisms of doxorubicin-induced cardiotoxicity?
2. How does Nrf2 participate in the pathological mechanisms of doxorubicin-induced cardiotoxicity?
3. How to ameliorate doxorubicin-induced cardiotoxicity through targeting Nrf2?

## Introduction

For decades, doxorubicin, a wide-ranging anticancer agent, has been extensively utilized in clinical settings to treat a variety of cancers due to its great therapeutic efficacy [1]. According to published studies, however, long-term administration of doxorubicin can cause severe damage to numerous organs in the body, with the heart being the primary target of doxorubicin toxicity [1, 2]. Long-term administration of doxorubicin can cause structural alterations in the heart, most notably reduced left ventricular ejection fraction,arrhythmias, reduced ventricular wall thickness, increased ventricular internal diameter and even heart failure [3–11]. According to the most recent epidemiological data, however, up to 5% of patients will exhibit varying degrees of cardiotoxic manifestations after doxorubicin administration, and the toxic effects become more severe with increasing cumulative doses, with the probability of heart failure reaching 48% when the cumulative dose reaches 700 mg/m^2^ [12, 13]. These severe cardiotoxic effects impose a considerable societal and familial economic and emotional burden.

**Fig. 1:**
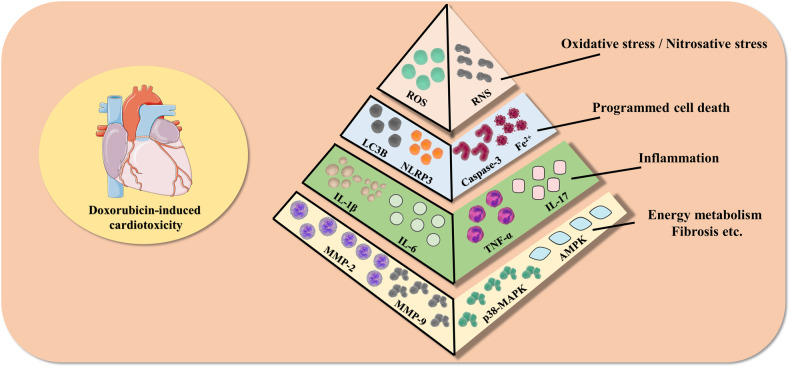
Pathological mechanisms of doxorubicin-induced cardiotoxicity. Produced using Servier Medical Art (smart.servier.com). Pathologic mechanisms of doxorubicin-induced cardiotoxicity include cardiomyocyte oxidative stress/nitrosative stress, programmed cardiomyocyte death (e.g., apoptosis, pyroptosis, autophagy, and ferroptosis), cardiomyocyte inflammation, and other mechanisms.

Many years of research have been devoted to determining the pathogenesis of doxorubicin-induced cardiotoxicity. Nevertheless, the specific etiology remains controversial, and the majority of the available research concentrates on the following factors. Firstly, it is well-established that oxidative stress/nitrosative stress in cardiomyocytes is the primary mechanism in doxorubicin-induced cardiotoxicity. It has been demonstrated that doxorubicin can trigger cardiac production of large quantities of reactive oxygen species (ROS) and reactive nitrogen species (RNS), while concurrently suppressing antioxidant mechanisms such as nuclear factor erythroid 2-related factor 2 (Nrf2). This is analogous to “double insurance” against cardiomyocyte damage [14, 15]. Likewise, there are studies on doxorubicin-induced cardiomyocyte programmed death. Several studies have demonstrated that doxorubicin can stimulate cardiomyocyte apoptosis by inducing ROS accumulation which results in mitochondrial damage, induce ferroptosis by regulating mitochondria and thereby promoting lipid peroxide and Fe^2+^ accumulation, inhibit cardiomyocyte autophagy, and induce cardiomyocyte pyroptosis by activating inflammasomes [14–20]. In addition, the findings of doxorubicin-induced inflammation in cardiomyocytes indicate that doxorubicin generates an inflammatory response by boosting the accumulation of inflammatory factors and nuclear expression of nuclear factor-κB (NF-κB) [21]. Of course, the pathogenesis of doxorubicin-induced cardiotoxicity goes far beyond those, as recent advances have revealed that doxorubicin can inhibit AMP-activated protein kinase (AMPK) and p38 mitogen-activated protein kinases (MAPK) energy metabolic pathways, thereby suppressing energy metabolism in cardiomyocyte to cause DNA damage [22, 23], and the pathway by which doxorubicin causes damage to cardiomyocyte has also been reported to be associated with cardiac fibrosis [24]. The pathological mechanism of doxorubicin-induced cardiotoxicity is shown in Fig. 1.

Nrf2 is a well-known antioxidant factor that plays a crucial function in mitigating oxidative stress [25]. However, available evidence indicates that it also plays a regulatory role in anti-cell programmed death and cell inflammation, as demonstrated by studies of doxorubicin-induced cardiotoxicity [26]. As described in studies pertaining to apoptosis, activation of Nrf2 can protect cardiomyocytes by inhibiting ROS expression levels and, consequently, reducing the mitochondrial apoptotic pathway in doxorubicin-induced cardiomyocytes [27]. In contrast, autophagy research has revealed that activation of Nrf2 can promote cell autophagy by regulating its downstream autophagy-related factors [28]. In the study of ferroptosis, it was also demonstrated that the Nrf2/glutathione peroxidase 4 (Gpx4) signaling pathway plays an essential role in regulating doxorubicin-induced ferroptosis in cardiomyocytes as a key regulatory pathway to inhibit lipid peroxidation [29]. In the study of pyroptosis, it was also found that regulation of Nrf2 expression substantially enhanced the modifications of pyroptosis-related proteins and reversed the doxorubicin-induced cardiomyocyte pyroptosis [30]. The Nrf2-mediated anti-inflammatory signaling pathway also plays a crucial role in cardiomyocyte inflammation [31].

In this review, we intend to emphasize the pivotal role of Nrf2 in doxorubicin-induced cardiotoxicity, in particular, the involvement of Nrf2 as a crucial mechanism in the pathology of doxorubicin-induced cardiotoxicity, as well as compounds that target Nrf2 for the treatment of doxorubicin-induced cardiotoxicity.

## The structure and regulation of Nrf2

### The structure of Nrf2

**Fig. 2:**
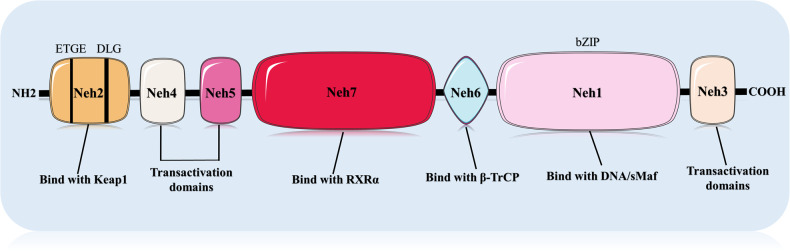
The basic structure diagram of Nrf2. Produced using Servier Medical Art (smart.servier.com). The basic structure of Nrf2 contains seven different structural domains, each of which plays a different function.

Nrf2 is a 66 kDa protein that is encoded by the NFE2L2 gene and belongs to the Cap‘n’collar (CNC) family of transcription factors [32, 33]. This protein consists of 605 amino acids and has seven highly conserved functional structural domains, including Nrf2-ECH homology 1 (Neh1)-Neh7 [32, 34, 35]. Neh1 is able to mediate the binding of Nrf2 to the Nrf2 antioxidant response element (ARE) in the nucleus, thereby promoting the transcription of various antioxidant enzymes, primarily due to the presence of the basic region leucine zipper (bZIP) gene sequence, which can bind to the small musculoaponeurotic fibrosarcoma (sMaf) protein [36]. Neh2 has two gene sequences, ETGE and DLG, that can interact with Kelch-like ECH-associated protein 1 (Keap1) and promote its ubiquitination [37]. Neh3, Neh4, and Neh5 interact to related proteins in order to increase ARE-dependent activation of associated genes [36, 38]. The Neh6 structural domain is serine-rich, binds to β-transducin repeat-containing protein (β-TrCP), and is linked to Keap1-independent Nrf2 negative regulation [39]. And, Neh7 can bind to retinoic X receptor (RXR), reducing Nrf2’s expression activity [39, 40]. Figure 2 depicts the basic structure diagram of Nrf2.

### The activation of Nrf2

**Fig. 3:**
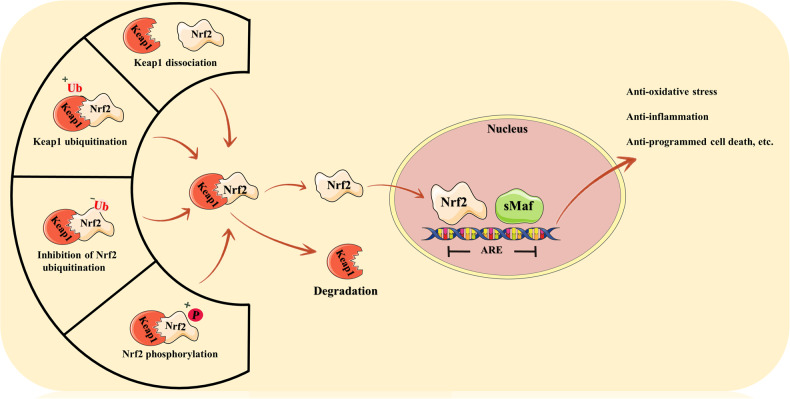
The activation of Nrf2. Produced using Servier Medical Art (smart.servier.com). The activation methods of Nrf2 include two major categories, the first one is related to Keap1, including the separation of Keap1, the ubiquitination of Keap1 and the reduced degradation ability of Keap1 to Nrf2 thus leading to the separation of Keap1 from Nrf2, allowing Nrf2 to enter the nucleus, and the second method is independent of Keap1, namely, the phosphorylation of Nrf2 on its own to achieve intranuclear migration.

The role of Nrf2 is dependent on its intranuclear migration to connect with other ARE-carrying genes, hence, the regulation of Nrf2’s intranuclear migration is the key to promoting its role [41]. We have detailed two types of approaches for regulating the nuclear translocation of Nrf2 based on the existing literature. The first is to “break the stranglehold” of Keap1, a specialized E3 ubiquitin ligase binding protein that functions as a significant “sensor” for the redox state of the cell and is a negative regulator of the nuclear translocation of Nrf2, and sometimes referred as a Nrf2 “inhibitor” [42, 43]. Under normal physiological conditions, Keap1 keeps Nrf2 in the cytoplasm and promotes Nrf2 breakdown by ubiquitination. However, in the presence of oxidative stress, Keap1 dissociates from Nrf2, resulting in intranuclear translocation of Nrf2 and a cascade of biochemical reactions [44]. The primary mechanisms of action of Keap1 activation of Nrf2 are hotly contested, but focus mostly on the three modes of action listed below. The first is the theory of Keap1 dissociation. It has been demonstrated that the cysteine residues in Keap1 can be modified, resulting in the separation of Nrf2 and Keap1 [44]. The “hinge and latch” notion of Keap1 is also pertinent. In academic circles, this view appears more far-reaching. It was found that Nrf2 can bind to Keap1 through a high-affinity ETGE gene sequence and a low-affinity DLG gene sequence, which are more like a “hinge” and a “latch.” However, in the presence of oxidative stress, the modification of cysteine residues in Keap1 leads to a change in Keap1 conformation, which seems to be more pronounced at the low-affinity “latch” site, resulting in the inability to ubiquitinate Nrf2 [45, 46]. The last is the Keap1 ubiquitination theory. The change of Nrf2 ubiquitination to Keap1 results in the destruction of Keap1 ubiquitination while Nrf2 is driven to detach [34]. The second type of strategy directly stimulates the phosphorylation of Nrf2, independent of Keap1. Certain kinases, including as c-Jun N-terminal kinase (JNK) and extracellular regulated kinase (ERK), are able to directly phosphorylate Nrf2 and induce its nuclear translocation to play a comparable role [47–50]. Figure 3 summarizes the activation of Nrf2.

### The function of Nrf2

The preceding part described how Nrf2 is triggered to reach the nucleus, and this section will describe Nrf2’s function within the nucleus. According to majority of research, the function of Nrf2 is intrinsically connected to oxidative stress. Research indicates that Nrf2 reaches the nucleus and attaches to AREs in antioxidant-related proteins such as heme oxygenase 1 (HO-1) via the “tracker” it carries, so stimulating the production of antioxidant components and generating antioxidant effects [25, 51]. It has been discovered, however, that Nrf2’s involvement is not limited to antioxidant effects alone. As stated in the introduction, Nrf2 also plays a key role in the regulation of programmed cell death, and this role is closely linked to mitochondria [52]. Nrf2 can affect apoptosis by regulating mitochondrial ROS expression, and ferroptosis by regulating mitochondrial accumulation of Fe^2+^, ROS and lipid peroxides [17, 27, 52–57]. And Nrf2 also plays a very crucial role in autophagy and mitophagy [28, 58]. And Nrf2 can also influence pyroptosis by affecting mitochondria [59, 60]. Moreover, by inhibiting inflammatory signaling pathways such as NF-κB, Nrf2 can also reduce the expression level of inflammatory factors, thereby exerting an anti-inflammatory effect [61, 62]. In addition, it has been demonstrated that Nrf2 can influence cell proliferation by activating cell proliferation-related factors such as IGF-1, and that Nrf2 expression has a crucial relationship with matrix reconstruction [25, 63]. Interestingly, mitochondrial biogenesis was also discovered to be closely related to Nrf2 [25, 64].

**Fig. 4:**
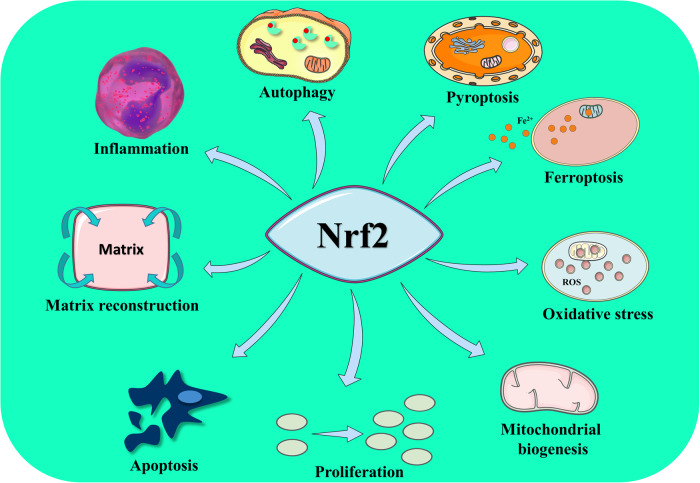
The function of Nrf2. Produced using Servier Medical Art (smart.servier.com). The role of Nrf2 is not only limited to antioxidant activity, but also plays an important role in apoptosis, pyroptosis, ferroptosis, autophagy, cell inflammation, matrix reconstruction, cell proliferation, and mitochondrial biosynthesis.

It is unquestionably true that Nrf2 plays a major role when it enters the nucleus. Consequently, future research should investigate Nrf2 with greater attention to detail. The functions of Nrf2 are summarized in Fig. 4.

## The role of Nrf2 in doxorubicin-induced cardiotoxicity

The pathogenesis of doxorubicin-induced cardiotoxicity is intricate and multifaceted, and Nrf2 is a critical regulator of this pathogenesis. Thus, this part will discuss the intimate connection between doxorubicin-induced cardiotoxicity and the Nrf2 signaling pathway.

### The role of Nrf2 in doxorubicin-induced oxidative stress in cardiomyocytes

Currently, oxidative stress is regarded as one of the trendiest topics in the whole medical field, but its definition is contested. In our opinion, oxidative stress should be defined as an imbalance between antioxidants and oxidants in the body, with the imbalance favoring the increase of oxidants, which leads to more ROS aggregation and body injury [65]. However, existing studies have also shown that ROS do not only act as a “behind the scenes” to damage cells, but that appropriate ROS can act as a second messenger to transmit signals to maintain redox homeostasis in the body, and this is the process of redox signaling [66–68]. In fact, it has been shown that redox is actually a precursor to oxidative stress [69, 70]. Under normal conditions, ROS in the body is in a dynamic equilibrium at a low level in which redox signaling plays a crucial role. However, when this equilibrium is disturbed, ROS accumulate, which disrupts the redox balance and causes oxidative stress [69]. And doxorubicin is a significant contributor to this imbalance [71–73]. According to the summary of existing studies, there are three main ways in which doxorubicin induces oxidative stress in cardiomyocytes by causing abnormal changes in ROS levels and thus disrupting redox signaling [24]. Initially, doxorubicin can lead to the accumulation of ROS by disrupting the ROS “production plant,” i.e., mitochondria. According to studies, doxorubicin can bind to cardiolipin in mitochondria to form a complex that is retained in the inner mitochondrial membrane, preventing the binding of related proteins to cardiolipin and producing high levels of ROS in cardiomyocytes [71]. Further, the details of oxidative stress in cardiomyocytes caused by disruption of iron metabolism by doxorubicin leading to iron overload are discussed in detail in the ferroptosis section of this chapter, along with a brief explanation of how disruption of iron homeostasis results in the formation of iron-doxorubicin (Fe-Dox) complexes that cause free radical cell damage [72]. However, the third one promotes ROS elevation by increasing NADPH oxidase, an essential redox marker. It has been demonstrated that doxorubicin treatment results in a very high level of NADPH oxidase, which in turn causes a significant increase in ROS levels in cardiomyocytes, thereby disrupting redox signaling and causing oxidative stress [73].

Long acknowledged as an antioxidant factor, Nrf2 has proved its high antioxidant potential in a number of disease-related research [25, 74, 75]. Recent research has demonstrated that the Nrf2 antioxidant pathway is closely associated with the development of doxorubicin-induced oxidative stress in cardiomyocytes. Nrf2 exerts its antioxidant effects primarily by entering the nucleus through the isolation of Keap1, where it binds to the ARE on antioxidant-related proteins such as HO-1 and NAD(P)H dehydrogenase quinone 1 (NQO1) [76]. Intriguingly, various upstream factors and pathways can influence the Nrf2-mediated oxidative stress signaling pathway, and it is likely that doxorubicin suppresses the intranuclear translocation of Nrf2 by regulating other upstream factors.

**Fig. 5:**
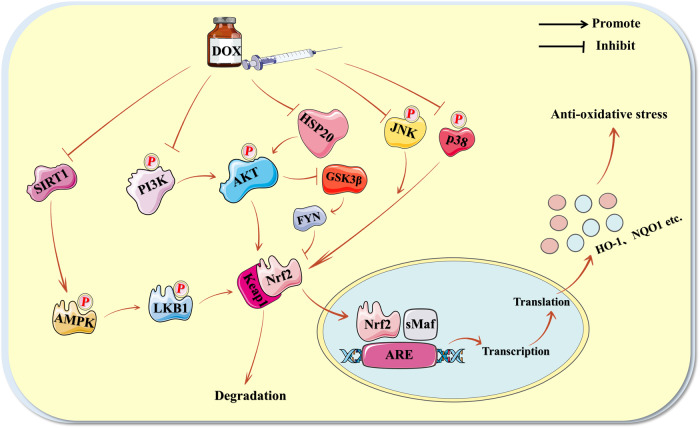
Specific molecular mechanisms of doxorubicin-induced oxidative stress in cardiomyocytes. Produced using Servier Medical Art (smart.servier.com). Doxorubicin can exacerbate the generation of oxidative stress in cardiomyocytes by inhibiting the nuclear translocation of Nrf2 through the inhibition of four different signaling pathways.

The primary finding is that the sirtuin-1 (SIRT1) signaling pathway can modulate the Nrf2-mediated antioxidant signaling pathway, which is identical to the apoptosis mechanism covered in the subsequent section. Recent studies have demonstrated that doxorubicin decreases the nuclear translocation of Nrf2 and the expression levels of its downstream antioxidant-related indicators, whereas SIRT1, a nicotinamide adenine dinucleotide (NAD^+^) dependent deacetylase. And SIRT1 downstream phosphorylated liver kinase B1 (p-LKB1) and p-AMPK are also affected by doxorubicin. Further, inhibitor experiments demonstrate that SIRT1 is an upstream regulator of Nrf2. Those indicate doxorubicin can induce oxidative stress in cardiomyocytes through inhibition of the SIRT1/LKB1/AMPK/Nrf2 signaling pathway [77–79]. Obviously, a more in-depth investigation of the SIRT1 signaling pathway revealed that its function is largely dependent on the signaling of AMPK and its downstream factors, including Nrf2 or the mammalian target of rapamycin (mTOR) signaling pathway [78]. In addition, the protein kinase B (AKT) signaling pathway can regulate the Nrf2 antioxidant signaling pathway, and the phosphatidylinositol-3 kinase (PI3K)/AKT signaling pathway is one of the most well-known signaling pathways. It has been demonstrated that doxorubicin can inhibit the Nrf2 antioxidant signaling pathway by regulating the PI3K/AKT signaling pathway, resulting in myocardial oxidative stress. Several studies have demonstrated that doxorubicin has a heightened sensitivity to oxidative stress indicators, specifically the inhibition of Nrf2 and its downstream antioxidant factors HO-1 and NQO1. This inhibition naturally extends to the expression of PI3K and AKT related proteins [31, 80]. Exploring further the link between PI3K/AKT and Nrf2, this experiment indicated that PI3K inhibitors can greatly diminish the nuclear translocation of Nrf2, indicating that PI3K/AKT is an upstream regulator of Nrf2 and that doxorubicin can induce myocardial oxidative stress by regulating PI3K/AKT for Nrf2 and its downstream antioxidant factors [80]. Existing investigations have demonstrated that in addition to the PI3K/AKT signaling pathway, heat shock protein-20 (HSP20)/AKT/glycogen synthase kinase 3 β (GSK3β)/FYN/Nrf2 plays a crucial role in doxorubicin-induced cardiac oxidative stress. This study initially demonstrated the stimulatory effect of doxorubicin on myocardial oxidative stress and the inhibitory effect of doxorubicin on the phosphorylation of AKT and other components. And further investigations were undertaken to investigate the relationship between this signaling axis. The observation that the use of AKT inhibitors in cardiomyocytes leads to activation of GSK3β, which in turn promotes activation of FYN, resulting in nuclear export and degradation of Nrf2, and that previous studies have demonstrated that HSP20 is closely associated with AKT activation [81–83]. This is sufficient to demonstrate that doxorubicin-induced oxidative stress in cardiomyocytes can occur through the HSP20/AKT/GSK3β/FYN/Nrf2 signaling pathway [81]. Lastly, there are studies that explore the MAPK signaling system, represented by JNK and p38, and the Nrf2 antioxidant signaling pathway in doxorubicin-induced oxidative stress in cardiomyocytes. As described above, these studies discovered the effect of doxorubicin on cardiomyocyte oxidative stress markers and the expression levels of Nrf2 and its downstream antioxidant proteins. Additionally, they demonstrated that the effect of doxorubicin on JNK and p38 phosphorylation could increase and then decreasing [54]. Further exploration of the association between the MAPK signaling pathway represented by JNK and p38 and the Nrf2 mediated antioxidant signaling pathway revealed that inhibition of the JNK and p38 signaling pathways in cardiomyocytes resulted in decreased nuclear expression of Nrf2 and instead increased Nrf2 expression levels in the cytoplasm, which demonstrates a close upstream and downstream connection between JNK and p38 and Nrf2, and it can be boldly speculated that the MAPK signaling pathway represented by JNK and p38 is an important control point for doxorubicin inhibition of the Nrf2 antioxidant signaling pathway [54, 84]. Figure 5 summarizes in detail the important role of Nrf2 in doxorubicin-induced oxidative stress in cardiomyocyte.

Doxorubicin-induced oxidative stress in cardiomyocytes is the main pathological mechanism responsible for cardiotoxicity, and interestingly, the available studies also suggest a possible “crosstalk” between oxidative stress and programmed cell death and cell inflammation in this process, which suggests that targeted oxidative stress therapy may cascade with treatment of programmed cell death and cell inflammation. However, whatever in oxidative stress or programmed cell death or cell inflammation, the Nrf2 is a ray hope certainly.

### The role of Nrf2 in doxorubicin-modulated autophagy in cardiomyocytes

In recent years, cell autophagy has been widely researched as a distinct sort of controlled death. Autophagy is a highly conserved cellular degradation process that isolates damaged organelles and cytoplasm into autophagosomes and transports them to “cellular degraders,” i.e. lysosomes, to form autophagic lysosomal complexes that degrade and recycle the available macromolecules produced by degradation [85]. And the AMPK and mTOR pathways are the two primary autophagy pathways. When the AMPK pathway is activated or the mTOR pathway is inhibited, the autophagosome is activated, while the light chain 3 (LC3) protein on the autophagosome membrane recruits LC3-interacting region (LIR)-containing proteins into the autophagosome and p62/Sequestosome 1 (SQSMT1) recruits ubiquitinated aggregates into the autophagosome as well, resulting in degradation of the damaged components [18, 86, 87]. However, mitochondria also play a significant role in autophagy. When inflammation, oxidative stress, and other stimulation are applied to cells, mitochondrial DNA is mutated, and simultaneously, the mitochondrial membrane potential is reduced and depolarization occurs in the cell, leading to cell death. In this case, the damaged mitochondria will “wrap” themselves up and degrade automatically to protect the cell, a process known as mitophagy [88, 89]. As a specific form of cell autophagy, the occurrence of mitophagy also involves two main pathways, namely the ubiquitin-dependent pathway associated with PTEN-induced kinase 1 (PINK1) and its E3 ubiquitin ligase Parkin (PRKN), and the non-ubiquitin-dependent pathway associated with the direct binding of LIR-containing proteins to LC3 [88, 90, 91].

Many factors affect the autophagy process, and the effect of the anthracycline doxorubicin on cellular autophagy has been extensively investigated in recent years. Many studies have demonstrated that doxorubicin influences the activation of the AMPK pathway, however more recent findings are inconsistent. Some studies have showed that doxorubicin can suppress autophagy by inhibiting AMPK activity [92–97]. However, others have demonstrated that doxorubicin has no effect on the AMPK pathway [98–102]. Nevertheless, doxorubicin has an effect not only on the AMPK pathway, but also on the mTOR pathway. Several studies have demonstrated that doxorubicin can inhibit the mTOR pathway, however, this inhibition has also been shown to cause cellular injury, which is highly controversial [98, 103–107]. Certainly, long-term doxorubicin treatment also influences the occurrence of mitophagy, which is primarily attributable to doxorubicin’s inhibitory effect on the mitochondrial ubiquitination-dependent pathway [108, 109].

Nrf2 has been in the public spotlight as an important target for the regulation of oxidative stress, but existing studies also suggest that Nrf2 may also be a key regulator of doxorubicin-modulated autophagy in cardiomyocytes. In a recent study, it was discovered that doxorubicin substantially decreased the expression level of Nrf2, while the expression level of p62/SQSMT1, which is a downstream factor of Nrf2 and represents the inhibition of autophagic flux, increased significantly. This indicates that doxorubicin can inhibit autophagic flux by inhibiting Nrf2 expression. This study probed deeper on the reasons for the inhibition of autophagic flux by doxorubicin and found that doxorubicin could alter the expression levels of transcription factor-EB (TFEB), a master transcription factor for lysosomal biogenesis, and lysosomal- associated membrane protein 1 (LAMP1), which demonstrated that the mechanism of doxorubicin to inhibit autophagy is through the inhibition of lysosomal biogenesis [28]. However, the role of doxorubicin in the regulation of Nrf2 is contentious. In a separate study, doxorubicin was found to substantially increase the expression levels of p62/SQSMT1 and ubiquitinated proteasome, which is ample evidence of doxorubicin’s detrimental effect on cellular autophagy. Nevertheless, the study also discovered that doxorubicin could increase Nrf2 expression levels. To demonstrate whether elevated levels of Nrf2 expression are beneficial, Nrf2 knockout mice were used in this experiment. By comparing the expression levels of autophagy as well as ubiquitinated proteasome before and after Nrf2 knockdown, it was demonstrated that elevated Nrf2 was beneficial for autophagy as well as clearance of ubiquitinated proteasome. Therefore, the authors hypothesize that the high expression of Nrf2 following doxorubicin induction is more of a “feedback” mechanism that inhibits the accumulation of ubiquitinated proteasomes that cause toxic reactions in the heart, thereby reducing the toxic effects of doxorubicin on the heart [110]. Notably, there is no direct evidence that Nrf2 plays a role in doxorubicin-modulated mitophagy. But one study suggests that phosphoglycerate mutase family member 5 (PGAM5) protein, a substrate of Keap1 along with Nrf2, is closely associated with mitophagy and that doxorubicin reduces its expression level to promote the binding of Parkin to PINK1 in mitochondria, thereby promoting mitophagy [17]. Since PGAM5 and Nrf2 are both Keap1 substrates, we may postulate as to whether they exert a synergistic or antagonistic effect, which may serve as a crucial link between Nrf2 and mitophagy in doxorubicin-induced cardiotoxicity.

In any case, the evidence suggests that Nrf2 can be regulated by doxorubicin and thus influencing the occurrence of autophagy in cardiomyocytes. However, there is still much to discuss, and this will be an important topic to investigate and debate in the future.

### The role of Nrf2 in doxorubicin-induced apoptosis in cardiomyocytes

Apoptosis is the most fundamental and extensively studied type of programmed cell death. It is essentially a specific type of cell injury, including chromatin condensation, fragmentation of the nucleus, and the formation of cell contraction and apoptotic vesicles, and it plays a crucial role in myocardial injury [111]. Among the various variables that trigger apoptosis, doxorubicin is a specific inducer of apoptosis. Previous research has described the primary methods by which doxorubicin produces apoptosis in cardiomyocytes, which may be categorized into two levels: the intrinsic apoptotic pathway and the extrinsic apoptotic pathway. The intrinsic apoptotic pathway (mitochondrial pathway) occurs because doxorubicin can disrupt the outer mitochondrial membrane of cardiomyocytes, resulting in the release of cytochrome c (Cyt C), which can activate caspase-3 by recruiting the production of caspase-9, and the activated caspase-3 can be transferred to the nucleus via the cytoplasm, thereby promoting DNA fragmentation [112]. The Fas-mediated extrinsic apoptotic pathway initiates apoptosis by activating Caspase-8, which in turn activates Caspase-3/7 [113], in contrast to the intrinsic apoptotic process, which mostly requires the activation of death receptors.

Although it is generally known that Nrf2 plays a crucial role in the field of antioxidants, recent research has also supported the significance of Nrf2 in cardiomyocyte apoptosis. According to research, excessively high or low levels of Nrf2 alters ROS levels, which in turn cause mitochondria to reduce membrane potential and ultimately cause apoptosis in cardiomyocytes [52, 57, 114–116]. However, doxorubicin has a significant role in the modification of Nrf2 levels [117–119]. Certainly, doxorubicin’s regulatory effect on Nrf2 is not always direct. Studies have shown that doxorubicin can also control Nrf2 via changing some of its upstream components, which causes the buildup of ROS and initiates apoptosis in cardiomyocytes.

Previously, it has been demonstrated that Nrf2 can be regulated by PI3K/AKT. As stated in the section on oxidative stress, the phosphorylated PI3K/AKT signaling pathway plays a crucial role in enabling the separation of Nrf2 from Keap1 in order to complete nuclear translocation. In a recent study, doxorubicin was discovered to greatly enhance the expression of Caspase-3 and BCL-2 associated X (Bax), while decreasing the expression of B cell lymphoma-2 (Bcl-2) in cardiomyocytes, indicating that doxorubicin can really alter apoptosis in cardiomyocytes [120]. Further experiments revealed that doxorubicin decreased the expression of Nrf2 and its downstream HO-1, glutathione cysteine ligase modulatory subunit (GCLM), and p-AKT, and that pharmacological intervention reversed the alterations of these factors, and that the expression levels of Nrf2 and its downstream indicators were once again reversed after AKT inhibition. Those demonstrate that PI3K/AKT is indeed the upstream pathway of Nrf2 and that doxorubicin can stimulate cardiomyocyte apoptosis by inhibiting PI3K/AKT through and thereby inhibiting the Nrf2 pathway [120]. However, we believe that the shortcoming of this study is the lack of detection of mitochondrial damage indicators and their membrane potential alterations, which is very crucial for further investigation of the pathways by which Nrf2 affects the onset of apoptosis in cardiomyocytes. And then, the p38 MAPK signaling pathway has also attracted a lot of interest in the study of cardiomyocytes as it is a crucial energy metabolic process. It has been shown that phosphorylation of p38 MAPK is protective for cardiomyocytes [121]. Nevertheless, a number of studies have shown that activation of p38 MAPK is associated with various pathological mechanisms of myocardial injury and that inhibition of p38 MAPK reduces cardiac fibrosis, cardiac hypertrophy, and oxidative stress in cardiomyocytes [121–123]. The controversy regarding the p38 MAPK signaling pathway is also present in doxorubicin-induced cardiotoxicity. It has been discovered that doxorubicin can promote the activation of p38 MAPK and thus inducing the activation of inflammatory signals such as NF-κB to promote the expression of inflammatory factors such as interleukin-1β (IL-1β), IL-6, IL-17 and tumor necrosis factor alpha (TNF-α) to induce inflammation, while inhibiting this pathway has the opposite effect [122, 124]. However, it has also been reported that doxorubicin induces cardiomyocyte apoptosis by inhibiting p38 MAPK and its downstream factor Nrf2. Recent research indicates that doxorubicin can substantially affect the phosphorylation of the p38 MAPK signaling pathway, and p38 MAPK inhibitors were reported to diminish the nuclear translocation of Nrf2 and its downstream antioxidant-related markers, such as HO-1 and paired related homeobox-1 (Prx1) expression levels, and to increase the number of apoptotic cells in cardiomyocytes [125]. In addition, a similar conclusion was found in a recent study. This experiment found that doxorubicin did lead to an increase in cardiomyocyte apoptosis, as evidenced by the number of apoptotic cells, the expression levels of Cyt C and the apoptosis-related factors Caspase-3, Caspase-9, Bax, and Bcl-2, and in an investigation of the relationship between p38 MAPK and Nrf2, it was also found that p38 MAPK inhibitors could inhibit Nrf2 activation. This demonstrates that p38 MAPK is indeed an upstream expression factor of Nrf2, and that doxorubicin can inhibit the Nrf2 signaling pathway through p38 MAPK, leading to myocardial apoptosis [54]. Additionally, it has been shown that there is also a close association between SIRT1 and Nrf2. In a recent study, doxorubicin was found to significantly increase the expression levels of apoptotic proteins Bax and Caspase-3 and significantly inhibit the expression levels of anti-apoptotic protein Bcl-2 in cardiomyocytes, and also significantly decrease the expression levels of nuclear translocation of Nrf2 and its downstream factors such as HO-1. And not only that, doxorubicin also affects the expression levels of SIRT1 and its downstream p-AMPK and p-LKB1 expression levels [77]. Further experiments were conducted to investigate how SIRT1 and Nrf2 are linked. They found that the expression levels of p-AMPK, p-LKB1 and Nrf2 were significantly reduced after silencing SIRT1 in the non-interfering group, suggesting that SIRT1 may be the “leader” of Nrf2 and it is likely that doxorubicin cause apoptosis by inhibiting SIRT1, thereby affecting the nuclear translocation of p-LKB1 and p-AMPK and thus inhibiting Nrf2 pathway [77]. A recent study also confirmed this conjecture and further investigated the link between SIRT1 and Nrf2 on the basis of this conjecture, and found that the relationship between SIRT1 and Nrf2 was not only upstream and downstream, but also SIRT1 was affected by silencing Nrf2, which proved that there was a significant “feedback” effect between SIRT1 and Nrf2 [27]. We discovered an intriguing aspect of the SIRT1 signaling pathway. Reviewing the literature, we discovered that there is also a connection between the SIRT1 signaling pathway and the previously mentioned MAPK signaling pathway, and the existence of this connection was demonstrated in cardiomyocytes. This study revealed that an increase in SIRT1 levels in cardiomyocytes could mitigate the onset of apoptosis by inhibiting the phosphorylation of p38 MAPK and JNK, while activating the phosphorylation of ERK [126]. For this reason, a new research direction comes to mind, namely, is the activation of Nrf2 related to the role of the SIRT1 signaling pathway in regulating the MAPK signaling pathway to exert an anti-apoptotic effect on cardiomyocytes? If related, is the pathological mechanism underlying doxorubicin-induced cardiomyocyte apoptosis associated with inhibition of SIRT1/MAPK/Nrf2? We believe that these are two future avenues worth investigating. Finally, one study found that Myheart (Mhrt), a myosin heavy chain-associated RNA, is also closely related to Nrf2 [127]. The effect of doxorubicin on apoptosis in cardiomyocytes is like the one described previously, but doxorubicin also affects the expression level of Mhrt and Nrf2, and to explore the relationship between Mhrt and Nrf2, this experiment overexpressed and silenced Mhrt, and it was found that the expression level of Nrf2 changed with the change of Mhrt, which proved the upstream and downstream relationship between Mhrt and Nrf2 [128]. Further experiments determined that Mhrt can regulate Nrf2 expression by prompting the Nrf2 promoter to bind to the H3 histone. This would suggest that the effect of doxorubicin on Nrf2 is likely to be achieved by first affecting the expression of Mhrt [128].

**Fig. 6:**
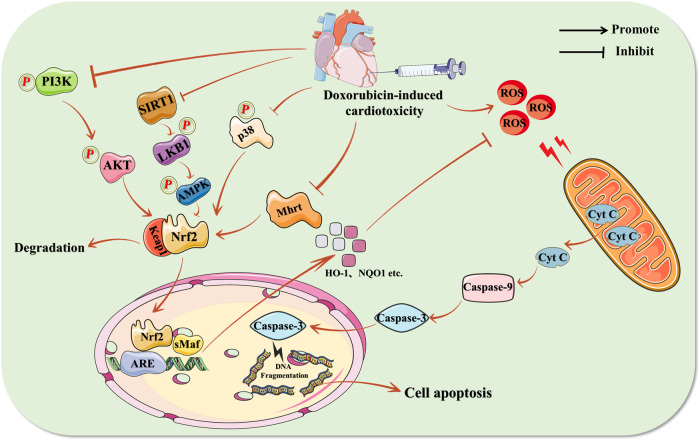
Specific molecular mechanisms of doxorubicin-induced apoptosis in cardiomyocytes. Produced using Servier Medical Art (smart.servier.com). Inhibition of antioxidant signaling pathways such as Nrf2 by doxorubicin leads to increasing levels of ROS in the body, which stimulates mitochondrial membrane damage in cardiomyocytes, leading to the production of apoptotic bodies, resulting in DNA fragmentation and thus leading to apoptosis in cardiomyocytes.

In conclusion, the apoptosis of cardiomyocytes induced by doxorubicin is closely related to the Nrf2 signaling pathway. Doxorubicin can elicit apoptosis by directly inhibiting the antioxidant capacity of Nrf2, resulting in a significant accumulation of ROS and mitochondrial damage. Doxorubicin also can inhibit Nrf2 by inhibiting factors upstream of Nrf2 to induce apoptosis in cardiomyocytes. In any case, Nrf2 must be a key target of doxorubicin-induced cardiomyocyte apoptosis. Consequently, selective modulation of Nrf2 may be a crucial method for inhibiting doxorubicin-induced cardiomyocyte apoptosis. Figure 6 summarizes in detail the key role played by Nrf2 in doxorubicin-induced apoptosis in cardiomyocytes.

### The role of Nrf2 in doxorubicin-induced ferroptosis in cardiomyocytes

Ferroptosis is a novel form of programmed cell death that is triggered by significant iron buildup and lipid peroxidation, and it is an essential mechanism for cellular injury [129]. Many causes can promote ferroptosis in cardiomyocytes. However, doxorubicin plays a crucial role in this process and has been intensively investigated in recent years. The mechanism of ferroptosis in cardiomyocytes caused by doxorubicin involves two aspects: initially, the disruption of iron homeostasis. Normally, circulating iron enters the body as Fe^3+^ by binding to transferrin (Tf) via transferrin receptor (TfR1), and the Fe^3+^ entering the body changed to Fe^2+^ and released into the cytoplasm via divalent metal transporter 1 (DMT1) [130]. One portion of Fe^2+^ is stored as a protein, one portion of Fe^2+^ is oxidized into Fe^3+^ and transferred outside the cell by ferritin transport protein (FPN), and the remaining portion is stored in the unstable iron pool [130]. And long-term doxorubicin intervention can result in a significant accumulation of iron, which can disrupt iron homeostasis and eventually cause ferroptosis. It has been discovered that doxorubicin can boost iron intake by altering homeostatic iron regulator (HFE) gene expression and, consequently, HFE-related protein production, thereby allowing HFE to bind to TfR1 to promote iron binding to Tf [131]. It has also been reported that doxorubicin maybe impact TfR1 receptors; however, this conclusion has not been confirmed. Moreover, it has been discovered that doxorubicin can influence iron metabolism by modulating iron-regulatory protein (IRP), resulting in iron accumulation [18, 132]. It was also discovered that doxorubicin could disrupt the permeability of mitochondria and alter the expression of the mitochondrial iron export protein ABC protein-B8 (ABCB8) protein in order to induce ferroptosis by causing iron accumulation within the mitochondria [131, 133].

The second element of ferroptosis in cardiomyocytes produced by doxorubicin is the promotion of lipid peroxide accumulation in cardiomyocytes. Doxorubicin can stimulate the accumulation of Fe^2+^, thereby inducing the Fenton reaction to stimulate the accumulation of lipid peroxides, which induces ferroptosis [134–136]. Moreover, doxorubicin induces the accumulation of significant quantities of DOX-Fe^2+^ complexes in mitochondria, which is a major source of lipid peroxidation accumulation [137–141]. Another target of doxorubicin to promote lipid peroxide accumulation is Nrf2. Doxorubicin can restrict the separation of Keap1 and Nrf2, thereby limiting the nuclear translocation of Nrf2, to inhibit the expression of its downstream anti-ferroptosis factors such as HO-1 and Gpx4, thereby permitting a decrease in glutathione synthesis, which results in the accumulation of lipid peroxides [29, 142, 143]. Nonetheless, the effect of doxorubicin on Nrf2 is still contested. A handful of studies have demonstrated that doxorubicin can overstimulate the activation of Nrf2, thereby promoting the expression of HO-1, which leads to the degradation of heme and the release of toxic substances such as Fe^2+^, which leads to a large accumulation of free iron in mitochondria to trigger lipid peroxidation damage [17, 144, 145]. Regardless, there is no doubt that doxorubicin induces ferroptosis in cardiomyocytes by regulating Nrf2. However, it was discovered that doxorubicin does not always regulate Nrf2 directly, but can also regulate Nrf2 by regulating its upstream factors. Currently, doxorubicin primarily regulates three important upstream factors to activate Nrf2 and induce ferroptosis in cardiomyocytes.

Initially, p62 can be involved in the Nrf2-mediated ferroptosis signaling pathway. Studies have shown that ferroptosis inducers can reduce the competitive binding of p62 protein to Keap1 by reducing its expression [146]. That allows Nrf2 to bind more tightly to Keap1 and thus decreasing the nuclear translocation expression of Nrf2, which further reduces the expression of downstream anti-cellular ferroptosis indicators such as Gpx4 [146]. In another study, administration of doxorubicin significantly reduced the expression levels of p62 with Nrf2 and its downstream anti-ferroptosis-related proteins HO-1 and Gpx4, thereby promoting cardiomyocyte ferroptosis, and p62 overexpression significantly increased the expression levels of Nrf2 and its downstream anti- ferroptosis-related proteins, demonstrating that doxorubicin can induce ferroptosis in cardiomyocytes by regulating p62 expression and thereby inhibiting Nrf2 expression [145, 146]. In addition, SIRT1, an important member of the NAD-dependent deacetylase family, plays a crucial role in mitochondrial damage and apoptosis mechanisms, but previous research has also identified it as an important upstream factor that can modulate the Nrf2-mediated anti-ferroptosis signaling pathway [147]. In one study, doxorubicin was found to drastically suppress the expression levels of SIRT1 protein and related Nrf2 as well as its downstream anti- ferroptosis markers such as HO-1 and Gpx4, so it is possible that SIRT1 is one of the key components of doxorubicin’s inhibition of Nrf2 that induces myocardial ferroptosis. By increasing the expression level of SIRT1 through pharmacological intervention, this hypothesis was confirmed. Inhibition of Nrf2 after activation of SIRT1 protein had no effect on the level of SIRT1, but a comparison of Nrf2 expression levels between activation and inhibition of SIRT1 demonstrated SIRT1 is an upstream regulator of Nrf2, and SIRT1/Nrf2 participates in doxorubicin-induced ferroptosis in cardiomyocytes [148]. However, existing studies have only demonstrated that SIRT1 can engage in the Nrf2-mediated ferroptosis signaling pathway, but the relationship between the two, i.e., how SIRT1 activates Nrf2, has not been conclusively established, and this issue must be investigated in future research. Finally, protein arginine methyltransferase 4 (PRMT4), a type I protein arginine methyltransferase, was discovered to be involved in the regulation of the Nrf2-mediated ferroptosis signaling system [149]. In a recent study, doxorubicin was found to significantly reduce the expression of Nrf2 and its downstream Gpx4 and nuclear receptor coactivator 4 (NCOA4), and this was exacerbated by PRMT4 overexpression. This led to the hypothesis that PRMT4 is involved in the Nrf2-mediated ferroptosis signaling pathway, and further studies demonstrated that PRMT4 can catalyze the methylation of Nrf2-associated enzymes, thereby restricting Nrf2 nuclear translocation. These indicate that PRMT4 is involved in doxorubicin’s inhibition of Nrf2-induced ferroptosis in cardiomyocytes [143].

In conclusion, Nrf2 can be an important indicator of doxorubicin-induced ferroptosis in cardiomyocytes, and targeting Nrf2 can effectively reduce doxorubicin-induced ferroptosis in cardiomyocytes. However, there are not enough studies in this area, and there are still need a large number of preclinical studies to support this, so future research should focus on this direction.

### The role of Nrf2 in doxorubicin-induced pyroptosis in cardiomyocytes

Cellular pyroptosis, an additional type of programmed cell death, was first postulated in 2001 and is characterized by the rapid breakdown of the plasma membrane and the rapid release of cellular contents and pro-inflammatory mediators, which play a significant role in myocardial injury [150, 151]. Interestingly, it has been demonstrated that cellular is closely related to cellular autophagy and redox signaling, and to be more specific, cellular pyroptosis can be controlled by redox signaling and autophagy. Although this is still debatable, it has been demonstrated that ROS generated by NADPH oxidase may act as redox signaling molecules to modulate NOD-like receptor family pyrin domain containing 3 (NLRP3) expression and activate cellular pyroptosis [152–157]. Activation of ROS has also been shown to directly cause gasdermin D-N (GSDMD-N) oligomerization, which induces cell pyroptosis by punching perforations in the cell membrane [158]. In investigating the relationship between autophagy and cellular pyroptosis, it was discovered that autophagy can modulate cellular pyroptosis in the opposite direction. These studies have focused on the “classical pathway” by which autophagy regulates cell pyroptosis in response to damage-associated molecular pattern molecules (DAMPs) and the “non-classical pathway” by which pyroptosis is regulated in response to pathogen-associated molecular pattern molecules (PAMPs) such as LPS, thereby achieving precise regulation of cell pyroptosis [159]. However, it is still unknown whether pyroptosis can affect redox reactions and autophagy, a topic that will require future research.

Being a key anticancer drug, doxorubicin has unquestionable advantages in the treatment of a variety of cancers. Nevertheless, investigations have indicated that long-term doxorubicin administration can result in cardiac harm, with myocardial cell pyroptosis being a key cause. Based on current research, the primary causes of doxorubicin-induced myocardial cell pyroptosis are “classical pathways” and “non-classical pathways.” The initial pathway can via the traditional pyroptosis pathway, also known as the Caspase-1 activation pathway. Doxorubicin stimulates the activation of tissue differentiation-inducing non-protein coding RNA (TINCR), which in turn induces a rise in insulin-like growth factor 2 mRNA-binding proteins (IGF2BP) and prepares for the activation of NLRP3 [160]. Massive activation of inflammasomes not only initiates inflammation, but also cellular pyroptosis. NLRP3 induces the activation of Caspase-1, which results in the cleavage of GSDMD-N, leading in the cleavage of cell membranes and the release of vast quantities of inflammatory chemicals, including IL-1 and IL-18 [161]. Existing studies have also demonstrated another pathway of doxorubicin-induced cardiomyocyte pyroptosis, namely the mitochondrial pathway, in which a large accumulation of doxorubicin leads to increased expression of Bcl-2/adenovirus E1B interacting protein 3 (Bnip3) protein in mitochondria, which activates caspase-3 to cause GSDMD protein, a protein that can punch holes in cell membranes, and the massive accumulation of this protein leads to disruption of cell membranes and thus promoting the resorption of cardiomyocytes [162, 163].

Recent research has showed that Nrf2 has a crucial role not only in oxidative stress, but also in apoptosis, ferroptosis, autophagy, and, of course, cell pyroptosis. In a recent study, it was discovered that doxorubicin drastically decreased the expression levels of Nrf2 and SIRT3 and considerably increased the expression of cell pyroptosis-related proteins such as Caspase-1, associated speck-like protein containing a CARD (ASC), GSDMD-N, and cytokines such as IL-1 and IL-18 [30, 59]. Further experiments involving overexpression of Nrf2 and SIRT3 revealed decreased expression levels of doxorubicin-induced myocardial pyroptosis-related proteins, indicating that the SIRT3/Nrf2 signaling pathway is involved in doxorubicin-induced myocardial cell pyroptosis [30]. As for how SIRT3 relates to Nrf2 to cause doxorubicin-induced cardiomyocyte pyroptosis, research reveals a link with doxorubicin-induced oxidative stress. They hypothesize that long-term doxorubicin treatment induces massive ROS production, which accumulates ROS and disrupts the expression of SIRT3 in mitochondria, thereby inhibiting the Nrf2 signaling pathway in cardiomyocytes and increasing the expression level of NLRP3 in order to activate the classical cell pyroptosis pathway [30].

Cardiomyocyte pyroptosis is an essential pathological mechanism of doxorubicin-induced cardiotoxicity, but it has not been sufficiently studied currently, and the role of Nrf2 as an important transcription factor against cardiomyocyte pyroptosis is not well understood, so this void should be investigated thoroughly in future research.

### The role of Nrf2 in doxorubicin-induced inflammation in cardiomyocytes

In practically all diseases, the inflammatory response is one of the oldest and most thoroughly researched underlying pathogenic mechanisms [164]. It is characterized by an imbalance in the coordination of anti-inflammatory and pro-inflammatory factors, which results in the buildup of excessive levels of pro-inflammatory substances that can cause harm to the organism [21]. Recent research has demonstrated that the pathogenic mechanism of cardiomyocyte damage in doxorubicin-induced cardiotoxicity is intimately connected to the inflammatory response. Doxorubicin has been reported to cause elevated expression of pro-inflammatory factors such as IL-1β, IL-8, and TNF-α in the cardiomyocyte, and NF-κB, a complex that controls the transcription of pro-inflammatory genes, is also regulated by doxorubicin. The regulation of NF-κB by doxorubicin mainly leads to myocardial cell inflammation by promoting the degradation of IkappaB (IκB) and thus promoting the intranuclear translocation of NF-κB and thus inspiring the activation of downstream pro-inflammatory indicators [61, 165].

In addition to the discussion of Nrf2’s crucial function in doxorubicin-induced programmed cell death and oxidative stress, it has been observed that doxorubicin-induced inflammation in cardiomyocyte may also be strongly connected with Nrf2. In a recent study, nuclear NF-κB p65 expression levels were increased in cardiomyocytes after doxorubicin intervention and promoted the expression of the pro-inflammatory factor IL-8. In addition, doxorubicin intervention also reduced the expression levels of Nrf2 and its upstream PI3K/AKT, while further experiments showed that NF-κB p65 expression levels increased and IL-8 expression levels increased after PI3K/AKT or Nrf2 inhibition, suggesting that the PI3K/AKT/Nrf2 signaling pathway may regulate the NF-κB p65 signaling pathway thereby regulating cardiomyocyte inflammation [31]. Where the p38/ NF-κB p65 signaling pathway has been shown to play an important role in doxorubicin-induced cardiomyocyte inflammation [166], therefore suggesting that the pathway of doxorubicin-promoted cardiomyocyte inflammation is likely to be associated with inhibition of the PI3K/AKT/Nrf2/p38/NF-κB p65 axis [31].

However, too few studies have been published on the promotion of myocardial inflammation by doxorubicin via inhibition of the Nrf2 signaling pathway, and the existing studies have primarily focused on the relationship between the pro-inflammatory effect of doxorubicin and NF-κB p65, while ignoring the connection with Nrf2. Thus, we appeal that future research should place a greater emphasis on the interaction between Nrf2 and NF-κB p65.

## Targeting Nrf2 for doxorubicin-induced cardiotoxicity

**Fig. 7:**
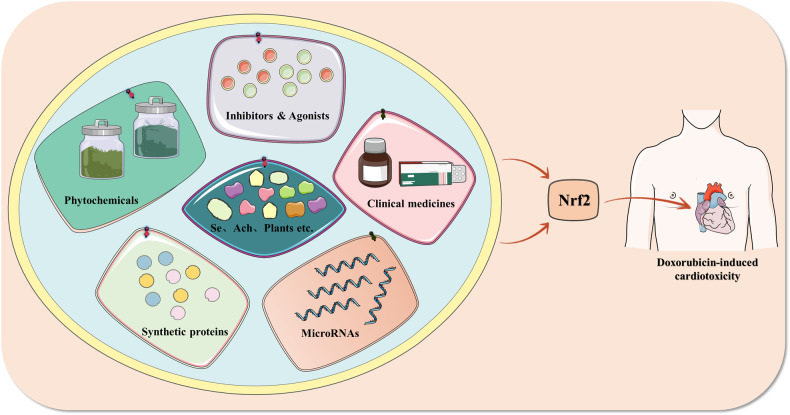
Compounds target the Nrf2 signaling pathway to treat doxorubicin-induced cardiotoxicity. Produced using Servier Medical Art (smart.servier.com). Many clinical drugs, natural compounds such as phytochemicals as well as others and synthetic compounds such as inhibitors and agonists, synthetic proteins, and others and some MicroRNAs can ameliorate doxorubicin-induced cardiotoxicity by targeting the Nrf2 signaling pathway.

The preceding mechanistic analysis demonstrates that the pathogenesis of doxorubicin-induced cardiotoxicity is closely linked to the inhibition of the Nrf2 signaling pathway, therefore, targeting the activation of the Nrf2 signaling pathway is an essential treatment strategy for doxorubicin-induced cardiotoxicity. Existing research suggests that a variety of clinical drugs, numerous natural and synthetic compounds, and MicroRNAs can activate the Nrf2 signaling pathway, which will be discussed in detail in this article. Figure 7 integrates the multiple activators that target Nrf2 to improve doxorubicin-induced cardiotoxicity. Table 1 summarizes all activators that can target Nrf2 to ameliorate doxorubicin-induced cardiotoxicity.

**Table 1:** Evidence for targeting Nrf2 to ameliorate doxorubicin-induced cardiotoxicity.

Category	Compound/protein/MicroRNA	In vivo/in vitro	Model (animals/cells)	Intervention parameters		Impact on Nrf2 signaling pathway	Reference
				Dose	Route		
Clinical medicines	Dimethyl fumarate	In vivo	DOX-induced SD rats	40, 80 mg/kg, twice a day	PO	Nuclear Nrf2, HO-1, NQO1, Bcl-2 expression ↑ ; Bax expression↓	[168]
		In vitro	DOX-induced primary myocytes	10, 20 μM for 4 h	N/A		
	Propofol	In vitro	DOX-induced H9c2 cells	5, 10, 20 μM for 1 h	N/A	Nuclear Nrf2, Gpx4 expression↑	[29]
Phytochemicals—flavonoids	Quercetin	In vivo	DOX-induced SD rats	10, 25, 50 mg/kg for 7 weeks	PO	Nuclear Nrf2 expression↑	[169]
	Genistein	In vivo	DOX-induced 129/sv mice	5 mg/kg for 4 weeks	IP	Nuclear Nrf2, HO-1, NQO1 expression ↑ ; Erk, Bax, Cleaved caspase-3 expression↓	[170]
	Didymin	In vivo	DOX-induced BALB/c mice	2, 4, 8 mg/kg every day for 1 week	IP	Nuclear Nrf2, HO-1, GCLM, p-PI3K, p-AKT, Bcl-2 expression ↑ ; Bax, Caspase-3/9 expression↓	[171]
		In vitro	DOX-induced H9c2 cells	5, 10, 20 µg/ml for 4 h	N/A		
	Cardamonin	In vivo	DOX-induced C57BL/6J mice	20, 40, 80 mg/kg/day	IG	Nuclear Nrf2, HO-1, NQO1, GCLM, Bcl-2 expression ↑ ; Bax, Cleaved caspase-3 expression↓	[172]
		In vitro	HL-1 cells	12.5–100 μM	N/A		
	Baicalein	In vivo	DOX-induced BALB/c mice	25, 50 mg/kg for 15 days	PO	Nuclear Nrf2, HO-1, NQO1 expression ↑ ; Bax/Bcl-2, Cleaved caspase-3, Cleaved PARP expression↓	[173]
	Isoorientin	In vivo	DOX-induced ICR mice	7.5, 15, 30 mg/kg for 8 times	IP	Nuclear Nrf2, Bcl-2 expression ↑ ; Cleaved caspase-3 expression↓	[174]
		In vitro	DOX-induced H9c2 cells	2.5–320 μg/ml	N/A		
	Pinocembrin	In vivo	DOX-induced C57BL/6J mice	5 mg/kg	IP	Nuclear Nrf2, SIRT3 expression ↑ ; NLRP3, ASC, Cleaved caspase-1, GSDMD-N, IL-18 expression↓	[30]
		In vitro	DOX-induced H9c2 cells	1 μM for 48 h	N/A		
	Acacetin	In vivo	DOX-induced C57BL/6J mice	15 mg/kg for 2 times/day for 3 days	SC	Nuclear Nrf2, HO-1, p-AMPK, p-LKB1, SIRT1 expression ↑ ; Bax, Cleaved caspase-3 expression↓	[77]
		In vitro	DOX-induced H9c2 cells	0.1–3 μM	N/A		
	Fisetin	In vivo	DOX-induced Wistar rats	20, 40 mg/kg/day	IG	Nuclear Nrf2, HO-1, Gpx4, FTH, FPN, FTL, SIRT1 expression↑	[148]
		In vitro	DOX-induced H9c2 cells	40 μM for 24 h	N/A		
Phytochemicals—polyphenols	Punicalagin	In vitro	DOX-induced H9c2 cells	50, 100, 200 μM for 2 h	N/A	Nuclear Nrf2, HO-1 expression ↑ ; Bax/Bcl-2 expression↓	[176]
	Tert-butylhydroquinone	In vivo	DOX-induced CD1/ICR mice	25 mg/kg/day for 3 days	IP	Nuclear Nrf2, HO-1, NQO1 expression↑	[177]
	Tanshinone I	In vivo	DOX-induced C57BL/6J mice	5, 10 mg/kg for 4 weeks	PO	Nuclear Nrf2, HO-1, NQO1, p-AKT expression↑	[178]
		In vitro	DOX-induced H9c2 cells	10 μM for 24 h	N/A		
	Resveratrol	In vivo	DOX-induced C57BL/6J mice	30 mg/kg for 12 weeks (cumulative dose to 210 mg/kg)	IP	Nuclear Nrf2, HO-1, NQO1, Gpx4, SLC7A11, p62 expression↑	[146]
		In vitro	DOX-induced H9c2 cells	0.5, 1 μM for 0.5 h	N/A		
	Dillenia pentagyna (Roxb)	In vivo	DOX-induced Balb/c mice	100 mg/kg for 2 days OR 200 mg/kg for 5 days	PO	Nuclear Nrf2, HO-1 expression↑	[179]
		In vitro	DOX-induced H9c2 cells	3.125, 6.25, 12.5, 25, 50 μg/ml for 24 h	N/A		
	Resveratrol	In vivo	DOX-induced C57BL/6J mice	10 mg/kg/day	IP	Nuclear Nrf2, HO-1, SIRT1 expression↑	[27]
		In vitro	DOX-induced H9c2 cells	20 μM	N/A		
	Yellow wine	In vivo	DOX-induced SD rats	30 mg/kg/day for 4 weeks	IP	Nuclear Nrf2 expression ↑ ; Bax/Bcl-2, Caspase-3 expression↓	[180]
		In vitro	DOX-induced H9c2 cells	50 mg/l	N/A		
Phytochemicals—saponins	Dioscin	In vivo	DOX-induced C57BL/6J mice DOX-induced SD rats	0, 20, 40 mg/kg/day for 5 days; 3, 4, 10 mg/kg/day for 7 days	PO	Nuclear Nrf2, HO-1, NQO1, MicroRNA-140-5p expression↑	[182]
		In vitro	DOX-induced H9c2 cells	0–1000 ng/ml for 24 h	N/A		
	Astragaloside IV	In vivo	DOX-induced SD rats	10 mg/kg/day for 5 weeks	IG	Nuclear Nrf2, Gpx4 expression ↑ ; NOX2, NOX4, Ptgs2 expression↓	[142]
Phytochemicals—terpenoids	Pritimerin	In vivo	DOX-induced Wistar rats	0.5, 1 mg/kg	IP	Nuclear Nrf2, HO-1, NQO1, GCLM expression↑	[165]
	Geraniol	In vivo	DOX-induced SD rats	200 mg/kg/day for 15 days	PO	Nuclear Nrf2, HO-1 expression↑	[183]
	Limonin	In vivo	DOX-induced SD rats	5, 10 mg/kg for 10 weeks	IP	Nuclear Nrf2, HO-1, NQO1, GCLM expression ↑ ; Bax/Bcl-2, Caspase-3 expression↓	[184]
		In vitro	DOX-induced H9c2 cells	1, 5, 25 μM for 48 h	N/A		
	Nerolidol	In vivo	DOX-induced Wistar rats	50 mg/kg for 5 weeks	IG	Nuclear Nrf2, HO-1, p-PI3K, p-AKT, Bcl-2 expression ↑ ; Bax, Caspase-3/9 expression↓	[80]
	Mokko lactone	In vivo	DOX-induced Wistar rats	15, 30 mg/kg	PO	Nuclear Nrf2, HO-1, Bcl-2 expression ↑ ; Bax, Caspase-3 expression↓	[185]
	Curdione	In vitro	DOX-induced H9c2 cells	12.5–150 mM	N/A	Nuclear Nrf2, HO-1, Bcl-2 expression ↑ ; Bax, Caspase-3, p-JNK, p-ERK expression↓	[186]
	Nerolidol	In vivo	DOX-induced Wistar rats	50 mg/kg for 5 days	PO	Nuclear Nrf2, HO-1, Bcl-2 expression ↑ ; Bax, Caspase-3/9 expression↓	[187]
	Asiatic acid	In vivo	DOX-induced Wistar rats	5, 10, 20 mg/kg for 7 days	PO	Nuclear Nrf2 expression↑	[188]
Phytochemicals—alkaloids	Tetrandrine	In vivo	DOX-induced C57BL/6J mice	50 mg/kg for 4 days	N/A	Nuclear Nrf2 expression ↑ ; Bax, Cleaved caspase-3 expression↓	[189]
		In vitro	DOX-induced NRCM cells	N/A			
	Rutaecarpine	In vivo	DOX-induced C57BL/6J mice	20 mg/kg for 4 weeks	N/A	Nuclear Nrf2, HO-1, p-AKT, GCLM expression ↑ ; Cleaved caspase-3 expression↓	[120]
Phytochemicals— sulfur-containing compounds	Sulforaphane	In vivo	DOX-induced 129/sv mice	4 mg/kg for 5 days/week	N/A	Nuclear Nrf2, HO-1 expression ↑ ; Caspase-3 expression↓	[190]
		In vitro	DOX-induced H9c2 cells	2.5 μM for 12–14 h	N/A		
	Sulforaphane	In vitro	DOX-induced H9c2 cells	N/A	N/A	Nuclear Nrf2, HO-1 expression ↑ ; Bax, expression↓	[191]
Synthetic proteins	Orosomucoid 1	In vitro	DOX-induced H9c2 cells	10, 30, 50, 100 mol	N/A	Nuclear Nrf2, HO-1 expression ↑ ; Cleaved caspase-3 expression↓	[192]
	Follistatin-like 1	In vitro	DOX-induced NRCM cells	5 μg/ml	N/A	Nuclear Nrf2, Bcl-2 expression ↑ ; Bax, Caspase-3 expression↓	[193]
	Obestatin	In vivo	DOX-induced SD rats	100 mg/kg/day for 4 weeks	IP	Nuclear Nrf2, Mhrt expression ↑ ; Caspase-3 expression↓	[128]
	Klotho	In vivo	DOX-induced SD rats	0.01 mg/kg for twice	IP	Nuclear Nrf2, HO-1, Prx1 expression↑	[125]
		In vitro	DOX-induced primary myocytes	400 pmol/l	N/A		
	Fibroblast growth factor-2	In vitro	DOX-induced ventricular cardiomyocytes	10 ng/ml for 30 min	N/A	Nuclear Nrf2, HO-1, p62/SQSTM1 expression ↑ ; Cleaved caspase-3, Bnip-3, p53 expression↓	[28]
	Fibroblast growth factor-21	In vivo	DOX-induced 129S1/SvImJ mice	100 μg/kg	IP	Nuclear Nrf2, HO-1, NQO1, CAT expression ↑ ; Caspase-9, Bax/Bcl-2 expression↓	[79]
		In vitro	DOX-induced H9c2 cells	50 ng/ml for 2 h	N/A		
	Kininogen-1	In vitro	DOX-induced H9c2 cells	NA	N/A	Nuclear Nrf2, HO-1 expression↑	[194]
	Octreotide	In vivo	DOX-induced SD rats	20 µg/kg	IP	Nuclear Nrf2 expression↑	[195]
Inhibitors and activators	ADP355	In vivo	DOX-induced C57BL/6J mice	200 ml/day for 4 weeks	IP	Nuclear Nrf2, HO-1, NQO1 expression ↑ ; Bax/Bcl-2 expression↓	[14]
		In vitro	DOX-induced H9c2 cells	N/A	N/A		
	Cilostazol	In vivo	DOX-induced ICR mice	50 mg/kg for 5 days/week for 6 weeks	IP	Nuclear Nrf2, HO-1, NQO1 expression↑	[196]
	Dapagliflozin	In vivo	DOX-induced SD rats	0.1 mg/kg for 5 days/week for 5 weeks	PO	Nuclear Nrf2, HO-1, NQO1, PI3K, AKT expression ↑ ; Bax/Bcl-2, NF-κB, IL-8 expression↓	[31]
	Rosiglitazone	In vivo	DOX-induced Wistar rats	10 mg/kg for 21 days	IG	Nuclear Nrf2, HO-1, NQO1, Bcl-2 expression↑	[197]
MicroRNAs	MicroRNA-200a	In vitro	DOX-induced H9c2 cells	50nmol/l	N/A	Nuclear Nrf2, HO-1 expression ↑ ; Bax expression↓	[200]
	MicroRNA -152	In vitro	DOX-induced NRCM cells	50nmol/l for 48 h	N/A	Nuclear Nrf2, HO-1, NQO1 expression↑	[201]
	MicroRNA-140-5p	In vivo	DOX-induced C57BL/6J mice	5nmol/g/day for 4 days	N/A	Nuclear Nrf2, HO-1, NQO1 expression↑	[198]
Other compounds	β-LAPachone	In vivo	DOX-induced C57BL/6J mice	2.5, 5 mg/kg for 3 days	IG	Nuclear Nrf2, HO-1, SIRT1, p-AMPK, p-LKB1 expression↑	[78]
	Naringenin-7-*O*-glucoside	In vitro	DOX-induced H9c2 cells	10, 20, 40 μM for 24 h	N/A	Nuclear Nrf2, HO-1, NQO1, GCLM expression↑	[84]
	Chitosan oligosaccharides	In vivo	DOX-induced SD rats	10, 30, 50, 70, 100 mg/kg for 2 weeks	IG	Nuclear Nrf2, HO-1, NQO1, Bcl-2 expression ↑ ; p-JNK, p-ERK Caspase-3, Caspase-9, Bax expression↓	[54]
		In vitro	DOX-induced H9c2 cells	100 μg/ml	N/A		
	Resolvin D1	In vivo	DOX-induced C57BL/6J mice	5.30 μg/kg for 1 min	IP	Nuclear Nrf2, HO-1, NQO1, Bcl-2 expression ↑ ; Caspase-3, Caspase-9, Bax expression↓	[202]
	Choline	In vivo	DOX-induced SD rats	7 mg/kg/day	IP	Nuclear Nrf2, HO-1, Bcl-2 expression ↑ ; Caspase-3, Bax expression↓	[203]
	α-Linolenic acid	In vivo	DOX-induced SD rats	500 mg/kg	IG	Nuclear Nrf2, Bcl-2 expression ↑ ; Caspase-3, Bax expression↓	[204]
	Tanshinone IIA	In vivo	DOX-induced mice	15, 30 mg/kg	IP	Nuclear Nrf2, HO-1, NQO1 expression↑	[76]
		In vitro	DOX-induced H9c2 cells	1, 3, 5, 10 μM	N/A		
	Danshensu	In vivo	DOX-induced KM mice	45 mg/kg/day for 3 days	IP	Nuclear Nrf2, HO-1, NQO1, Bcl-2 expression ↑ ; Caspase-3, Bax expression↓	[205]
	Hemin	In vivo	DOX-induced Wistar rats	2.5, 5, 10 mg/kg for 7 days	IP	Nuclear Nrf2, HO-1 expression ↑ ;	[206]
	Shengmai	In vivo	DOX-induced SD rats	2.7, 5.4, 10.8 ml/kg for 7 days	IP	Nuclear Nrf2, HO-1, Bcl-2 expression ↑ ; Caspase-3/9, Bax expression↓	[207]
		In vitro	DOX-induced H9c2 cells	2 ml for 24 h	N/A		
	Dimethyl itaconate	In vivo	DOX-induced C57BL/6J mice	100 mg/kg for 4 days	IP	Nuclear Nrf2, HO-1 expression↑	[208]
	Ganoderma lucidum polysaccharides	In vivo	DOX-induced C57BL/6J mice	50, 100 mg/kg	PO	Nuclear Nrf2, HO-1, Bcl-2 expression ↑ ; Bax expression↓	[209]
		In vitro	DOX-induced H9c2 cells	N/A	N/A		
	Selenium	In vivo	DOX-induced C57BL/6J mice	0.2 mg/kg for 2 weeks	PO	Nuclear Nrf2 expression ↑ ; NLRP3 expression↓	[59]

*DOX* doxorubicin, *PO* per os, *IP* intraperitoneal injection, *IG* intragastrical administration, *SC* subcutaneous injection, *N/A* not applicable.

### Activation of Nrf2 by clinical drugs ameliorate doxorubicin-induced cardiotoxicity

Clinical drugs have been the center of our attention. Moreover, evidence suggests that the mechanism of action of a number of clinical drugs involves modulation of the Nrf2 signaling pathway. First, it has been demonstrated that Propofol, a common intravenous anesthetic in modern medicine, inhibits the cardiotoxic effects of doxorubicin by modulating the nuclear translocation of Nrf2, primarily by inhibiting ferroptosis in cardiomyocytes, oxidative stress, and the onset of apoptosis in cardiomyocytes [29, 167]. In addition to Propofol, Dimethyl Fumarate, a first-line treatment for severe psoriasis and multiple sclerosis, performs a crucial role in the treatment of doxorubicin-induced cardiotoxicity. Dimethyl Fumarate substantially reduces the cardiomyocyte-damaging effects of doxorubicin, as evidenced by serum CK, LDH, and the reduction in cardiomyocyte apoptosis and oxidative stress levels detected in biochemical assays [168]. Unfortunately, only a limited number of studies have focused on clinical drugs to reduce doxorubicin-induced cardiotoxicity by targeting Nrf2, so we believe this is a promising area for future development and investigation.

### Activation of Nrf2 by natural compounds ameliorate doxorubicin-induced cardiotoxicity

Numerous natural compounds, phytochemicals included, serve a crucial role in activating Nrf2 in order to mitigate doxorubicin-induced cardiotoxicity. According to research, the following phytochemical categories can activate Nrf2 and reduce doxorubicin-induced cardiotoxicity. The first is flavonoids. Existing research indicates that flavonoids, such as quercetin, genistein, didymin, and others, can ameliorate doxorubicin-induced cardiotoxicity by reversing the inhibition of nuclear translocation of Nrf2 by doxorubicin and promoting the nuclear expression of Nrf2, which can activate downstream antioxidant-related indicators or inhibit pyroptosis, ferroptosis, and apoptosis indicators, thereby mitigating the incidence of cardiotoxicity [30, 77, 148, 169–175]. Not only may flavonoids mitigate doxorubicin-induced cardiotoxicity by increasing Nrf2 expression, but polyphenols can also exert this effect. Researchers discovered that polyphenols such as punicalagin, tert-butylhydroquinone, and resveratrol, etc. play an essential role in promoting Nrf2 nuclear translocation in doxorubicin-inhibited cardiomyocytes, regulating upstream factors of Nrf2, and promoting the expression of Nrf2 downstream regulators [27, 146, 176–180]. Other types of phytochemicals, such as glycyrrhizic acid among saponins [142, 181, 182], nerolidol among terpenoids [80, 165, 183–188], β-LAPachone among quinones [78], tetrandrine among alkaloids [120, 189], and sulfur-containing compounds such as sulforaphane [190, 191], are present in a large number of studies reporting their ability to reduce oxidative stress and cardiomyocyte death in doxorubicin-induced cardiotoxicity by modulating the nuclear translocation of Nrf2 and thus promoting the expression of downstream antioxidant and other indicators.

### Activation of Nrf2 by synthetic compounds ameliorate doxorubicin-induced cardiotoxicity

Many synthetic compounds, in addition to clinical drugs and numerous phytochemicals, can mitigate doxorubicin-induced cardiotoxicity by regulating the Nrf2 signaling pathway. Acute phase proteins orosomucoid 1 and follistatin-like 1 reduce the adhesion of Nrf2 to Keap1 in doxorubicin-induced cardiomyocytes, thereby promoting the nuclear translocation of Nrf2 and reducing the excessive accumulation of ROS in cardiomyocytes, thereby exerting antioxidant and anti-apoptotic effects [28, 79, 125, 128, 192–195]. Numerous other agonists and inhibitors, such as lipocalin agonists and phosphatase inhibitors, have been demonstrated in the available literature to promote doxorubicin-induced nuclear expression of Nrf2 in cardiomyocytes, thereby reducing the generation of oxidative stress in cardiomyocytes to reduce damage to the mitochondrial membrane of cardiomyocytes and thereby ameliorating apoptosis and oxidative stress in cardiomyocytes [14, 31, 196, 197].

### Activation of Nrf2 by MicroRNAs ameliorate doxorubicin-induced cardiotoxicity

In addition to clinical drugs, natural molecules and synthetic compounds, there are many more substances, such as MicroRNAs, that perform the same function. Several MicroRNAs, including MicroRNA-140-5p, MicroRNA-200a, MicroRNA-24-3p, and MicroRNA-152, are implicated in doxorubicin-induced oxidative stress and programmed cell death in cardiomyocytes, according to existing research. These studies found that MicroRNAs such as MicroRNA-200a could reduce ROS accumulation by targeting Keap1 mRNA and thus causing Keap1 mRNA degradation, thus promoting the intranuclear translocation of Nrf2, as well as promoting the improvement of mitochondrial function and activating the expression of downstream antioxidant and anti-apoptotic factors [198–201]. Obviously, we think that there is still much to be discovered in the field of RNA, and it is worthwhile to investigate whether many of its small molecule RNAs, such as long noncoding RNAs (LncRNA) and circular RNAs (CircRNA), also play a role in doxorubicin-induced cardiotoxicity.

In summary, there are a large number of natural or synthetic compounds that do not fall into the categories listed above and are thus not mentioned in the text. The compounds are described in the table below. To mitigate doxorubicin-induced cardiotoxicity, however, clinical drugs and natural compounds, as well as synthetic compounds and small molecule RNAs, play a crucial part in targeting Nrf2.

## Future and prospect

Compared to other clinical anti-cancer agents, doxorubicin has a broader anti-tumor spectrum, as a result, it has received increased attention in cancer treatment. The most recent epidemiological data indicates, however, that long-term doxorubicin treatment may cause irreparable heart harm, as suggested by research. The area of doxorubicin-induced cardiotoxicity that has received the most attention is currently pathogenesis research. Many studies have revealed that oxidative stress is the most important mechanism in the pathology of doxorubicin-induced cardiotoxicity, and that this pathology, which is accompanied by the production of high amounts of damaging ROS, causes irreparable damage to cardiomyocytes. In addition to oxidative stress, other complicated mechanisms, including cardiomyocyte programmed cell death, cardiomyocyte inflammation, and obstruction of cardiomyocyte energy metabolism, are crucial pathogenic causes of doxorubicin-induced cardiotoxicity. Nrf2 has been described as a unique endogenous regulator of anti-oxidative stress, and indeed, it has been discovered that its function is not limited to antioxidant capacity, but also includes anti-cell death and anti-cell inflammation. Consequently, the question of whether Nrf2 plays a significant role in doxorubicin-induced cardiotoxicity has generated a great deal of controversy and several research findings. As hypothesized, Nrf2 does play a key role in doxorubicin-induced cardiotoxicity, and it can be stated that the pathophysiology of doxorubicin-induced cardiotoxicity is mostly related to Nrf2 suppression. In this regard, research on how to target Nrf2 to mitigate doxorubicin-induced cardiotoxicity has become a hot topic, and numerous studies have demonstrated that numerous clinical drugs, natural compounds, synthetic compounds, and even numerous small molecule RNAs can ameliorate doxorubicin-induced cardiotoxicity by activating the Nrf2 signaling pathway. Until now, after reviewing the progress of earlier studies, we discovered that there are still numerous areas of research in this subject that require improvement. Initially, it was determined that the overall experimental design of the available studies was relatively cursory, whereas deeper processes exist in the relationship between Nrf2 and the pathogenesis of doxorubicin-induced cardiotoxicity that require further study. In addition, the majority of the literature on the pathogenesis of Nrf2 in doxorubicin-induced cardiotoxicity relates to cardiomyocyte oxidative stress, cardiomyocyte death, and cardiomyocyte inflammation, but not to other pathogenic mechanisms such as energy metabolism and endoplasmic reticulum stress, so we believe this is a research gap that needs to be filled with a substantial amount of study. Moreover, the majority of approaches to target Nrf2 for the treatment of doxorubicin-induced cardiotoxicity have centered on compound therapy, and many natural plant compounds have been studied more extensively. We are in favor of this direction of research, but one concern is that many plant compounds are dose-effective and toxic, and if not properly controlled, can lead to increased cardiac damage. Hence, we offer a novel concept, namely, if the recently studied and non-toxic aerobic exercise treatment can be employed as an intervention for Nrf2 to treat doxorubicin-induced cardiotoxicity, which is a previously unexplored field with a great deal of untapped potential. Ultimately, we appeal that Nrf2 engineered mice can serve as the primary subject in preclinical research. The majority of existing in vivo studies have used Nrf2 inhibitors and other methods to investigate Nrf2 at a deeper level, and only a few studies have used Nrf2 engineered mice as experimental subjects. However, compared to engineered mice, inhibitors and other methods have many limitations in terms of manipulation. Therefore, we believe that Nrf2 engineered mice may be superior in this regard.
